# 24-h movement behaviors from infancy to preschool: cross-sectional and longitudinal relationships with body composition and bone health

**DOI:** 10.1186/s12966-018-0753-6

**Published:** 2018-11-26

**Authors:** Rachael W. Taylor, Jillian J. Haszard, Kim A. Meredith-Jones, Barbara C. Galland, Anne-Louise M. Heath, Julie Lawrence, Andrew R. Gray, Rachel Sayers, Maha Hanna, Barry J. Taylor

**Affiliations:** 1 Departments of Medicine, Dunedin School of Medicine, University of Otago, PO Box 56, Dunedin, New Zealand; 2 Departments of Women’s and Children’s Health, Dunedin School of Medicine, University of Otago, Dunedin, New Zealand; 3 Departments of Human Nutrition, Dunedin School of Medicine, University of Otago, Dunedin, New Zealand; 4 Departments of Biostatistics Unit, Dunedin School of Medicine, University of Otago, Dunedin, New Zealand; 5 Departments of Office of the Dean, Dunedin School of Medicine, University of Otago, Dunedin, New Zealand

**Keywords:** Physical activity, Sedentary behavior, Sleep, Children, Compositional time use

## Abstract

**Background:**

New physical activity guidelines for children address all movement behaviors across the 24-h day (physical activity, sedentary behavior, sleep), but how each component relates to body composition when adjusted for the compositional nature of 24-h data is uncertain.

**Aims:**

To i) describe 24-h movement behaviors from 1 to 5 years of age, ii) determine cross-sectional relationships with body mass index (BMI) z-score, iii) determine whether movement behaviors from 1 to 5 years of age predict body composition and bone health at 5 years.

**Methods:**

24-h accelerometry data were collected in 380 children over 5–7 days at 1, 2, 3.5 and 5 years of age to determine the proportion of the day spent: sedentary (including wake after sleep onset), in light (LPA) and moderate-to-vigorous physical activity (MVPA), and asleep (including naps). BMI was determined at each age and a dual-energy x-ray absorptiometry (DXA) scan measured fat mass, bone mineral content (BMC) and bone mineral density (BMD) at 5 years of age. 24-h movement data were transformed into isometric log-ratio co-ordinates for multivariable regression analysis and effect sizes back-transformed.

**Results:**

At age 1, children spent 49.6% of the 24-h day asleep, 38.2% sedentary, 12.1% in LPA, and 0.1% in MVPA, with corresponding figures of 44.4, 33.8, 19.8 and 1.9% at 5 years of age. Compositional time use was only related significantly to BMI z-score at 3.5 years in cross-sectional analyses. A 10% increase in mean sleep time (65 min) was associated with a lower BMI z-score (estimated difference, − 0.25; 95% CI, − 0.42 to − 0.08), whereas greater time spent sedentary (10%, 47 min) or in LPA (10%, 29 min) were associated with higher BMI z-scores (0.12 and 0.08 respectively, both *p* < 0.05). Compositional time use from 1 to 3.5 years was not related to future BMI z-score or percent fat. Although MVPA at 2 and 3.5 years was consistently associated with higher BMD and BMC at 5 years, actual differences were small.

**Conclusions:**

Considerable changes in compositional time use occur from 1 to 5 years of age, but there is little association with adiposity. Although early MVPA predicted better bone health, the differences observed had little clinical relevance.

**Trial registration:**

ClinicalTrials.gov number NCT00892983.

**Electronic supplementary material:**

The online version of this article (10.1186/s12966-018-0753-6) contains supplementary material, which is available to authorized users.

## Background

Three key behaviors which increase the risk of obesity in children are low levels of physical activity, excess time spent sedentary, and inadequate sleep [1–3]. While it is clear that each of these behaviors have independent effects on health, they also interact in ways that may not be apparent if studied individually [3, 4]. However, the majority of research has examined the effects of physical activity, sedentary behavior, and sleep in isolation [5]. This is flawed because time spent in one behavior will naturally depend on the composition of the rest of the day due to the finite nature of the 24-h window: if one component increases (e.g. sleep), then another component within the same 24-h block *must* decrease (e.g. sedentary time or physical activity or both) [4]. This means that 24-h movement data require different methods of analysis than standard multivariate techniques to take into account the co-dependence and proportional nature of compositional data [6, 7].

Despite the release of 24-h movement guidelines for preschool children in several countries [8, 9], relatively few studies have collected 24-h movement data and even fewer have used appropriate compositional techniques to evaluate relationships with health [10–13]. Studies in older children and adolescents have demonstrated that each individual movement component (e.g. sleep, sedentary behavior) is independently related to BMI or fitness [11–13]. In the single study undertaken in preschool aged children, the overall composition of movement behaviors was related to BMI z-score, but not each individual component relative to the other components [10].

To date, all studies are cross-sectional in nature, which limits clarification of the benefits of replacing time in one behavior with another [3]. Only two studies appear to have used 24-h accelerometry to assess all components of movement patterns [11, 12]; the others have used accelerometry data to measure physical activity and sedentary time with questionnaires [10] or time diaries [13] to estimate sleep duration. However, it is unlikely that questionnaires are sensitive enough to detect the marked night-to-night variability in sleep duration that is revealed when 24-h accelerometry analyses are used in children [14]. No studies have investigated 24-h movement behaviors in infants and toddlers using compositional analyses, although a small body of work has examined adherence to new physical activity guidelines [15–17]. Similarly, none have used more accurate measures of adiposity than BMI to determine relationships with compositional time use, nor investigated other facets of body composition such as bone health [3].

Our recent Prevention of Overweight in Infancy (POI) study collected 24-h accelerometry data on a large sample of children for one week at 1, 2, 3.5 and 5 years of age, as well as accurate measures of body composition including bone health at 5 years of age. Therefore, the aims of this analysis were to i) describe 24-h movement behaviors from 1 to 5 years of age, ii) determine cross-sectional relationships between 24-h movement behaviors and body mass index (BMI) z-score, and iii) determine whether movement behaviors from 1 to 5 years of age predict body composition and bone health at 5 years.

## Methods

Data from the POI randomised controlled trial were used. POI was an obesity prevention initiative investigating whether anticipatory guidance to parents about infant sleep, food, activity, and breastfeeding resulted in different growth patterns compared with usual care in children followed from before birth to 5 years of age. As the intervention did not produce evidence for differences in physical activity, sedentary behavior, or sleep [18–20], these analyses have used the entire cohort with adjustment for randomisation group.

As detailed information on the original trial is available in both the study protocol [21] and published findings [19, 20], only brief details are provided here. The original intervention was approved by the Lower South Regional Ethics Committee (LRS/12/08/063) and the follow-up study by the University of Otago Human Ethics Committee (12/274). Written informed consent was obtained from the parent/guardian of all child participants. All mothers who had booked into the single maternity hospital (> 97% of all births) serving the population of Dunedin, New Zealand were invited to participate when in mid to late pregnancy (May 2009 to December 2010). A 58% response rate yielded a final sample size of 802 primiparous (47.6%) and multiparous (52.4%) mothers. All anthropometric assessments and accelerometry analyses were performed by researchers blinded to group allocation.

### Measures obtained at baseline

Demographic information obtained at baseline (late pregnancy) included maternal age, education, ethnicity, self-reported pre-pregnancy height and weight, and level of household deprivation [22]. Information on infant gestational age, sex and birth weight was obtained from hospital records.

### Measures obtained at 1, 2, 3.5 and 5 years of age

Anthropometric measurements were obtained by trained research staff following standard protocols [23] at each age. Duplicate measures of weight (Tanita WB-100 MA/WB-110 MA) and height (Harpenden stadiometer, Holtain Ltd., UK) were obtained with children wearing underwear/light clothing [24]. Quality control testing showed inter-rater reliability was excellent at each time point (> 0.93). BMI z-scores were determined using the WHO growth standards [25], with overweight defined as a BMI z-score ≥ 85th but <95th percentile, and obesity as BMI ≥ 95th percentile.

Physical activity and sleep were assessed with children wearing Actical (Mini-Mitter, Bend, OR) accelerometers 24-h a day for up to 7 days at each age. The accelerometers were worn around the waist on elastic belts and initialized using 15 s epochs. Data were cleaned and scored using an automated script developed in MATLAB (MathWorks, Natick, MA, USA) that uses a count-scaled algorithm to estimate sleep onset and offset for overnight sleep, awakenings (wake after sleep onset, WASO) and daytime naps specific to each individual for each day [26, 27]. Once these sleep data are effectively “removed” from the 24-h day, time spent sedentary and at various intensities of activity can be determined. Non-wear time was defined as at least 20 min of consecutive zeros during awake time data only [28], which provides acceptable estimates of physical activity (*r* = 0.70–0.90) in young children [29, 30]. As Actical cutoffs for physical activity intensity categories do not exist that span this full age range (1–5 years), we defined sedentary time as 0–25 counts (per 15 s), light physical activity (LPA) as 26–697 counts, and moderate-to-vigorous physical activity (MVPA) as ≥698 counts [31], based on validation work in toddlers and preschoolers [32–34]. Data from our group shows that Actical accelerometers worn around the waist provide a valid measure of sleep duration in young children when compared against polysomnography (Meredith-Jones, unpublished). At 1 year of age, parents were asked whether the infant could walk alone (defined as “baby takes at least five steps independently in an upright position - there is no contact with a person or object”). All analyses at 1 year of age used this measure to adjust for whether or not the child was walking.

### Measures obtained at 5 years of age only

Children underwent one total body DXA scan to assess body composition at 5 years of age only. DXA scans were performed and analysed by one experienced operator using a Lunar Prodigy scanner (GE Medical Systems Lunar, Madison WI); standard scanning procedures were used [35] and scans were analysed using the Lunar software package, version 16.0, with the pediatric option selected. Body composition variables assessed were percent body fat (fat mass as a percentage of body weight), bone mineral content (BMC, g) and bone mineral density (BMD, g/cm^2^). Both bone measures were total body less head (TBLH) as recommended in children [36].

### Statistical analyses

#### Normalising to 24 h

In order to keep each overnight sleep as a solid block of data, each 24-h ‘day’ was determined from the time the child woke up on day 1, to the time they woke up on day 2 (and so on). However, this means that each ‘day’ does not add up to 1440 min (as it might if midnight to midnight periods were used for example). A day was thus considered valid if the participant had between 20 and 28 h of data to allow for changing wake times across different days. Currently, there is no consensus on what represents a valid data when examining 24-h data. While others [11] have used 16 h a day (for at least 3 days), we believe this had the potential to include a lot of non-wear time given 16 h represents just 67% of a 24-h day. Our use of at least 20 h (83% of a 24-h day) was chosen to provide greater confidence in minimising non-wear time. In our 24 h data, non-wear time was less than 5%. This meant that the sleep and activity components had to be normalised to 24 h. Before this, the components during awake day time (naps; sedentary, light, moderate and vigorous activity) were normalised for all awake day time (including non-wear time), on the assumption that non-wear time (such as device removal for bathing) did not occur overnight when children were asleep. Otherwise, simply ignoring non-wear time would essentially allocate, incorrectly, some of the non-wear time to night-time sleep and time awake after sleep onset. Normalisation was undertaken by multiplying the proportion of time spent in each component for the ‘day’ by 1440 min (24 h).

### Weighting for weekend and weekday data

Participants needed to have at least three valid days of data to be included, which could have included a mix of weekdays and weekend days. Weekdays were weighted so that all weekdays together had a weight of 5/7, and weekend days were similarly weighted so that all weekend days had a weight of 2/7, making the data representative of a full week.

### Descriptive statistics

Both arithmetic and compositional means were calculated for each component. Compositional means are found by taking the geometric means for each of the components and dividing by the sum of all component geometric means, then normalising to 24 h by multiplying by 1440 min. Standard deviations of compositional means are not appropriate, instead the variances of the log ratios between the components were calculated. This is a measure of how proportional two components are – values closer to zero have a higher degree of proportionality.

### Isometric log-ratio coordinates

Due to the closed nature of the 24-h movement data, perfect multicollinearity exists and therefore traditional multivariable statistical methods are inappropriate. Recent studies into the compositional nature of 24-h movement and BMI outcomes have recommended the use of isometric log-ratio (ilr) coordinates, where the transformation of the data is well-described [7, 12]. Because log-ratios become problematic with zeros, time spent in day time naps and wake after sleep onset (time spent awake in the night between sleep onset and sleep offset) were reallocated to time spent asleep and sedentary, respectively. A set of ilr coordinates was calculated for each component of the day (i.e. a set for each of sleep, sedentary, light and MVPA). As there were four components in the day, there were four sets of ilr coordinates, with three coordinates in each set.

### Associations between 24-h movement patterns and body composition and bone health

Separate regression models were used for each set of ilr coordinates (e.g. the sleep set). The regression coefficient of the first ilr coordinate was used to describe the relationship between the time component (e.g. sleep) and the outcome (e.g. BMI z-score) because the first ilr coordinate is proportional to the log ratio of the component time and the geometric mean of the remaining component times. This regression coefficient can be back-transformed to give a meaningful estimate of the strength of the association. Estimates are described in terms of “time reallocation”. This terminology is used because the predictor variable is a function of the ratio between component time (the numerator) and remaining component times (the denominator). Therefore a “unit change” in the predictor variable is a measure of how much time from the particular component is reallocated to time in all other components (i.e. from the numerator to the denominator or vice versa). We used the methods as described by Dumuid et al. [12] to give the estimated difference in the outcome variable (e.g. BMI z-score) associated with a 10% difference in time allocation to a specific component (e.g. sleep). As the relationship is non-linear, an estimated difference for both a 10% greater and a 10% lower time allocation was calculated, along with 95% confidence intervals for this estimate. A 10% difference in time allocation was chosen in preference to the use of a set number of minutes (e.g. 15, 30 etc) to allow consistency in the size of the effect examined; 15 min of MVPA for example represents a much larger proportion of total daily MVPA than 15 min of sleep does for total daily sleep.

Linear regression models were used to determine cross-sectional, longitudinal and prospective associations all adjusted for randomised group, sex and demographic variables (and walking status for BMI z-score analyses at 1 year of age). BMI z-score at all ages, and percent body fat, and total body less head BMD (g/m^2^) and BMC (g) at 5 years of age were the outcome variables. The longitudinal associations with BMI z-score were adjusted for concurrent BMI z-score. The prospective bone analyses were further adjusted for BMI z-score given the known differences in bone relating to body size in children. Residuals were plotted and visually assessed for normality and homogeneity of variance. Missing data was excluded list-wise. All data were analysed using Stata 15.1 (StataCorp, Texas).

## Results

Table 1 presents the characteristics of the study population at baseline (birth) from the original POI study, and those with available data for analyses. Overall, mothers were predominantly New Zealand European (84.1%), and two-thirds (67.2%) had a University education. Approximately half the group were having their first child (48.3%), and fewer children came from homes with higher levels of neighbourhood deprivation than is seen nationally (i.e. 16.5% compared to the expected 30%).

**Table 1:** Demographic characteristics of the study population at baseline

		All POI participants	Those with at least one set of data^a^ n (%)	Those with at least one set of data^a^ and DXA at 5 years n (%)
n		802	380	257
*Maternal variables*				
Age at child’s birth (y)	*Mean (SD)*	31.6 (5.2)	32.5 (4.7)	33.2 (4.4)
Ethnicity n (%)	New Zealand European	682 (85.1)	299 (78.7)	216 (84.1)
	Māori	46 (5.7)	36 (9.5)	18 (7.0%)
	Other	73 (9.1)	45 (11.8)	23 (9.0)
	*Missing*	*1*	*2*	*0*
Parity n (%)	Primiparous	382 (47.6)	187 (49.2)	124 (48.3)
	Multiparous	420 (52.4)	193 (50.8)	133 (51.8)
Education^b^ n (%)	School only	193 (24.1)	78 (20.6)	48 (18.8)
	Post-secondary	116 (14.6)	50 (13.2)	36 (14.1)
	University degree or higher	485 (61.1)	250 (66.1)	172 (67.2)
	*Missing*	*8*	*2*	*1*
Pre-pregnancy BMI (kg/m^2^)	*Mean (SD)*	25.1 (5.0)	25.4 (5.1)	25.3 (5.2)
*Household or infant variables*				
Household deprivation^c^	1–3 (Low)	276 (34.8)	140 (37.0)	102 (40.0)
	4–7	350 (43.6)	171 (45.2)	111 (43.5)
	8–10 (High)	168 (21.2)	67 (17.7)	42 (16.5)
	*Missing*	*8*	*2*	*2*
Infant sex	Male	391 (48.8)	195 (51.3)	125 (48.6)
	Female	411 (51.2)	185 (48.7)	132 (51.4)
Infant birth weight (kg)	*Mean (SD)*	3551 (480)	3576 (492)	3581 (496)

Data presented as n (%) unless otherwise indicated

^a^At least 3 days of accelerometry at 1, 2, 3.5 or 5 years of age

^b^Secondary schooling in New Zealand is from year 9 to year 13 inclusive, ‘post-secondary qualifications’ refer to all tertiary qualifications that are not University based

^c^Uses the New Zealand Index of Deprivation 2013 which combines nine variables from the 2013 census relating to communication (no access to the internet at home), income (receiving a means tested benefit or living below income thresholds), unemployment, qualifications, home ownership, single parent families, living space, and transport access. A deprivation score is provided for each meshblock which is a geographical unit defined by Statistics New Zealand containing about 60–110 people. The score reflects the extent of material and social deprivation and is used to construct deciles from 1 (least deprived) to 10 (most deprived)

Table 2 presents the characteristics of participants at each point who had valid accelerometry data. Children who provided at least one set of valid accelerometry data (*n* = 380) compared to children providing no accelerometry data at any age (*n* = 422) had mothers who were older at birth (32.5 years vs 30.7 years, *p* < 0.001), and more highly educated (66.1% had a tertiary education compared to 56.5%, *p* = 0.019), but were not different according to ethnicity (*p* = 0.722) pre-pregnancy BMI (*p* = 0.090), household deprivation (*p* = 0.070), infant sex (*p* = 0.970) or birthweight (*p* = 0.162).

**Table 2:** Characteristics of the group at each time point including allocation of time use

Variable	Category	Age (years)			
		1	2	3.5	5
n		355	213	231	248
Female n (%)		172 (48.5)	93 (43.7)	107 (46.3)	122 (49.2)
Age (months)		12.2 (0.3)	24.1 (0.2)	41.9 (0.5)	59.7 (0.4)
Height (cm)		75.6 (2.8)	86.2 (3.1)	99.1 (3.8)	109.9 (4.4)
Weight (kg)		9.8 (1.1)	12.5 (1.4)	16.0 (1.8)	19.4 (2.4)
BMI (kg/m^2^)		17.2 (1.3)	16.8 (1.3)	16.3 (1.2)	16.0 (1.2)
BMI z-score		0.39 (0.83)	0.70 (0.89)	0.61 (0.83)	0.47 (0.81)
Weight status^a^ n (%)	Normal weight	281 (79.2)	140 (65.7)	158 (68.4)	190 (76.6)
	Overweight	52 (14.7)	43 (20.2)	45 (19.5)	40 (16.1)
	Obese	22 (6.2)	30 (14.1)	28 (12.1)	18 (7.3)
Sleep components^b^	Night-time sleep	578 (55)	594 (60)	626 (44)	632 (39)
(minutes)	Wake after sleep onset	29 (27)	20 (22)	15 (16)	12 (14)
	Day time naps	121 (43)	89 (42)	23 (26)	-^c^
Activity components^b^	Sedentary	524 (58)	443 (61)	462 (54)	476 (58)
(minutes)	LPA	184 (64)	284 (56)	292 (54)	287 (54)
	MVPA	4 (6)	10 (8)	22 (14)	33 (17)

Data presented as mean (SD) except for weight status which is n (%) and only includes those with valid accelerometry data

LPA is light physical activity, MVPA is moderate-to-vigorous physical activity

^a^Normal weight defined as BMI < 85th, overweight as a BMI z-score ≥ 85th but <95th percentile, and obesity as BMI ≥ 95th percentile according to the WHO growth standards [25]

^b^Data presented as arithmetic mean (SD) which is the average number of minutes spent in each category after individualised normalisation to 24 h

^c^Could not be calculated as no children napped at this age

**Fig. 1:**
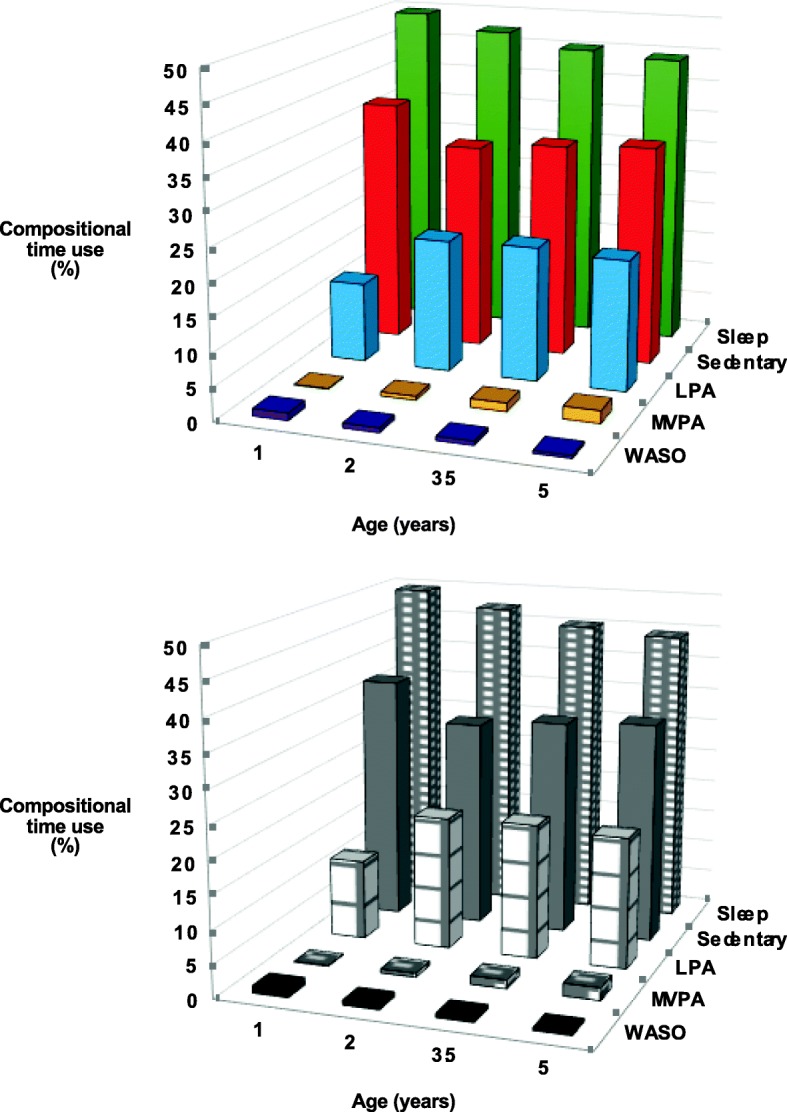
Compositional time use (% of 24-h day) at different ages. Data presented as compositional means - the geometric mean that has been re-normalised to 24 h (1440 min) for all component geometric means. Data presented as minutes (% of 1440 min) after normalisation to 24 h for each participant. Night-time sleep and naps have been combined. LPA is light physical activity, MVPA is moderate-to-vigorous physical activity, WASO is wake after sleep onset

Figure 1 demonstrates the differential time allocation from 1 to 5 years of age. At 1 year of age, infants spent nearly 50% of their 24-h day asleep (arithmetic mean of 699 min or 11.7 h including naps), almost 9 h being sedentary, about 3 h in light intensity activity, and virtually no time in MVPA. Marked changes occur between 1 and 2 years of age, with a substantial increase in light intensity activity in particular (100 min more per day on average). Over these four years, gradual increases in MVPA are observed, with children spending 33 min on average each day, 1.9% of the 24-h period, by 5 years of age. Additional file 1: Table S1 shows the variances of the log ratios, which indicate that time spent asleep, sedentary and in LPA had high proportionality, especially after 12 months of age.

No cross-sectional relationships were observed between compositional time use and BMI z-score at 1, 2 or 5 years of age (Table 3). Similarly, compositional time use was not related to BMI z-score when measured longitudinally (Table 4). However, at 3.5 years of age, spending 10% more time asleep (65 additional minutes) relative to other behaviors was associated with a lower BMI z-score (estimated difference, − 0.248: 95% CI -0.418 to − 0.080) whereas spending 10% more of the day in LPA (29 min) or being sedentary (47 min) was associated with higher BMI z-scores (0.122, 0.010 to 0.23 and 0.081, 0.015 to 0.147 respectively, Table 3). Tables 5 (lean mass) and 6 (percent fat mass) demonstrate that these differences in BMI reflect differences in lean mass rather than fat mass.

**Table 3:** Cross-sectional relationships between sleep and activity components and BMI z-score at each age^a^

Age (years)	Mean (SD) BMI z-score	Component	Regression coefficient^b^ (SE)	*p*-value	10% relative difference in minutes	Estimated difference (95% CI) in BMI z-score with a 10% *increase* in component	Estimated difference (95% CI) in BMI z-score with a 10% *decrease* in component
1 (*n* = 346)	0.39 (0.83)	Sleep^c^	0.00 (0.30)	0.989	71	−0.001 (− 0.099, 0.097)	0.001 (− 0.098, 0.099)
		Sedentary^d^	0.02 (0.30)	0.934	56	−0.008 (− 0.087, 0.072)	0.008 (− 0.074, 0.090)
		LPA	0.03 (0.14)	0.816	18	0.010 (−0.013, 0.033)	−0.011 (− 0.036, 0.014)
		MVPA	−0.06 (0.04)	0.130	0.1	−0.004 (− 0.010, 0.002)	0.004 (− 0.003, 0.012)
2 (*n* = 213)	0.70 (0.89)	Sleep	0.45 (0.40)	0.265	69	0.074 (−0.056, 0.204)	−0.075 (− 0.205, 0.056)
		Sedentary	−0.28 (0.37)	0.448	46	−0.035 (− 0.124, 0.055)	0.037 (− 0.058, 0.131)
		LPA	−0.08 (0.33)	0.804	28	−0.009 (− 0.076, 0.059)	0.009 (− 0.064, 0.082)
		MVPA	−0.09 (0.08)	0.285	0.6	−0.007 (− 0.020, 0.006)	0.008 (− 0.006, 0.022)
3.5 (*n* = 231)	0.61 (0.83)	Sleep	**−1.57 (0.55)**	**0.005**	**65**	**−0.248 (− 0.418, − 0.080)**	**0.252 (0.079, 0.425)**
		Sedentary	**0.97 (0.45)**	**0.034**	**47**	**0.122 (0.010, 0.235)**	**−0.129 (− 0.247, − 0.011)**
		LPA	**0.77 (0.32)**	**0.017**	**29**	**0.081 (0.015, 0.147)**	**−0.087 (− 0.158, − 0.016)**
		MVPA	− 0.17 (0.13)	0.182	2	−0.014 (− 0.036, 0.007)	0.016 (− 0.007, 0.039)
5 (*n* = 248)	0.44 (0.84)	Sleep	−0.89 (0.54)	0.100	64	−0.137 (− 0.300, 0.026)	0.140 (− 0.026, 0.306)
		Sedentary	0.25 (0.41)	0.534	49	0.032 (− 0.070, 0.135)	−0.034 (− 0.141, 0.073)
		LPA	0.55 (0.30)	0.072	28	0.057 (− 0.005, 0.118)	−0.061 (− 0.128, 0.005)
		MVPA	0.09 (0.13)	0.481	3	0.008 (−0.014, 0.029)	−0.008 (− 0.032, 0.015)

LPA is light physical activity, MVPA is moderate-to-vigorous physical activity

^a^Adjusted for randomised group, sex, primiparous, maternal education, household deprivation, ethnicity and maternal BMI. Analyses at 1 year of age further adjusted for whether the child was walking unaided or not walking

^b^Coefficient for the first isometric log ratio (ilr) co-ordinate, which is proportional to the log ratio of the behavior and the geometric mean of the remaining behaviors; adjusted for remaining ilr co-ordinates

^c^All sleep estimates include naps where relevant

^d^All sedentary estimates include wake after sleep onset

Bolded values indicate *P* < 0.05

**Table 4:** Predictive relationships between sleep and activity components at 1, 2 and 3.5 years of age, and *BMI z-score* at 5 years^a^

Age (years)	Mean BMI z-score (SD) at 5 years of age	Component	Regression coefficient^b^ (SE)	*p*-value	10% relative difference in minutes	Estimated difference (95% CI) in BMI z-score with a 10% *increase* in component	Estimated difference (95% CI) in BMI z-score with a 10% *decrease* in component
1 (*n* = 292)	0.43 (0.83)	Sleep^c^	− 0.24 (0.24)	0.336	71	−0.038 (− 0.119, 0.043)	0.038 (− 0.043, 0.119)
		Sedentary^d^	0.28 (0.25)	0.266	56	0.024 (−0.042, 0.089)	−0.024 (− 0.092, 0.043)
		LPA	−0.08 (0.12)	0.510	18	0.002 (−0.017, 0.021)	−0.002 (− 0.023, 0.019)
		MVPA	0.03 (0.03)	0.312	0.1	0.003 (−0.003, 0.008)	−0.003 (− 0.009, 0.003)
2 (*n* = 190)	0.43 (0.89)	Sleep	− 0.43 (0.28)	0.126	69	−0.071 (− 0.162, 0.020)	0.072 (− 0.020, 0.164)
		Sedentary	0.45 (0.26)	0.082	46	0.056 (−0.007, 0.120)	−0.059 (− 0.126, 0.007)
		LPA	−0.05 (0.24)	0.817	28	−0.006 (− 0.054, 0.042)	0.006 (− 0.046, 0.058)
		MVPA	0.03 (0.06)	0.559	0.6	0.003 (−0.007, 0.012)	− 0.003 (− 0.013, 0.007)
3.5 (*n* = 212)	0.44 (0.84)	Sleep	−0.08 (0.30)	0.784	65	−0.013 (− 0.107, 0.081)	0.013 (− 0.082, 0.108)
		Sedentary	−0.24 (0.25)	0.326	47	−0.031 (− 0.091, 0.030)	0.032 (− 0.032, 0.096)
		LPA	0.23 (0.17)	0.183	29	0.024 (−0.011, 0.060)	−0.026 (− 0.065, 0.012)
		MVPA	0.09 (0.07)	0.182	2	0.008 (− 0.004, 0.019)	−0.009 (− 0.021, 0.004)

LPA is light physical activity, MVPA is moderate-to-vigorous physical activity

^a^Adjusted for concurrent BMI z-score, randomised group, sex, primiparous, maternal education, household deprivation, ethnicity and maternal BMI. Analyses at 1 year of age further adjusted for whether the child was walking unaided or not walking

^b^Coefficient for the first isometric log ratio (ilr) co-ordinate, which is proportional to the log ratio of the behavior and the geometric mean of the remaining behaviors; adjusted for remaining ilr co-ordinates

^c^All sleep estimates include naps where relevant

^d^All sedentary estimates include wake after sleep onset

**Table 5:** Predictive and concurrent relationships between sleep and activity components at 1, 2, 3.5 and 5 years of age and *fat-free mass index* at 5 years^a^

Age (years)	Mean fat free mass index (SD) at 5 years of age, kg/m^2^	Component	Regression coefficient^b^ (SE)	*p*-value	10% difference in minutes	Estimated difference (95% CI) in % body fat with a 10% *increase* in component	Estimated difference (95% CI) in % body fat with a 10% *decrease* in component
1 (*n* = 229)	12.7 (0.8)	Sleep^c^	− 0.46 (0.32)	0.144	71	−0.079 (− 0.184, 0.026)	0.079 (− 0.027, 0.185)
		Sedentary^d^	0.27 (0.32)	0.393	56	0.038 (−0.049, 0.124)	−0.039 (− 0.129, 0.050)
		LPA	0.16 (0.15)	0.278	18	0.015 (−0.012, 0.043)	−0.017 (− 0.046, 0.013)
		MVPA	0.03 (0.04)	0.488	0.1	0.003 (−0.005, 0.010)	−0.003 (− 0.011, 0.005)
2 (*n* = 158)	12.8 (0.8)	Sleep	−0.32 (0.35)	0.365	69	−0.053 (− 0.166, 0.061)	0.053 (− 0.061, 0.168)
		Sedentary	0.60 (0.32)	0.065	46	0.075 (−0.004, 0.154)	−0.079 (− 0.162, 0.004)
		LPA	−0.37 (0.32)	0.244	28	−0.039 (− 0.104, 0.026)	0.042 (− 0.028, 0.112)
		MVPA	0.09 (0.08)	0.234	0.6	0.008 (−0.005, 0.020)	−0.008 (− 0.022, 0.005)
3.5 (*n* = 178)	12.8 (0.8)	Sleep	−**2.19 (0.53)**	**< 0.001**	**65**	**−0.346 (− 0.509, − 0.183)**	**0.352 (0.186, 0.518)**
		Sedentary	**0.87 (0.43)**	**0.046**	**47**	**0.110 (0.003, 0.217)**	**−0.116 (− 0.229, − 0.003)**
		LPA	**1.26 (0.31)**	**< 0.001**	**29**	**0.132 (0.070, 0.195)**	**−0.143 (− 0.210, − 0.075)**
		MVPA	0.06 (0.12)	0.641	2	0.005 (− 0.015, 0.024)	− 0.005 (− 0.027, 0.017)
5 (*n* = 213)	12.7 (0.8)	Sleep	−0.82 (0.55)	0.134	64	−0.127 (− 0.292, 0.039)	0.129 (− 0.039, 0.298)
		Sedentary	0.17 (0.41)	0.673	49	0.022 (−0.080, 0.124)	−0.023 (− 0.131, 0.084)
		LPA	0.48 (0.30)	0.116	28	0.050 (− 0.012, 0.111)	−0.054 (− 0.120, 0.013)
		MVPA	0.17 (0.13)	0.170	3	0.015 (− 0.006, 0.035)	−0.016 (− 0.039, 0.007)

LPA is light physical activity, MVPA is moderate-to-vigorous physical activity

^a^Adjusted for randomised group, sex, primiparous, maternal education, household deprivation, ethnicity and maternal BMI. Analyses at 1 year of age further adjusted for whether the child was walking unaided or not walking

^b^Coefficient for the first isometric log ratio (ilr) co-ordinate, which is proportional to the log ratio of the behavior and the geometric mean of the remaining behaviors; adjusted for remaining ilr co-ordinates

^c^All sleep estimates include naps where relevant

^d^All sedentary estimates include wake after sleep onset

Bolded values indicate *P* < 0.05

**Table 6:** Predictive and concurrent relationships between sleep and activity components at 1, 2, 3.5 and 5 years of age and *percent fat* at 5 years^a^

Age (years)	Mean % body fat (SD) at 5 years of age	Component	Regression coefficient^b^ (SE)	*p*-value	10% difference in minutes	Estimated difference (95% CI) in % body fat with a 10% *increase* in component	Estimated difference (95% CI) in % body fat with a 10% *decrease* in component
1 (*n* = 232)	15.6 (4.8)	Sleep^c^	− 1.24 (1.96)	0.528	71	−0.21 (− 0.86, 0.44)	0.21 (− 0.44, 0.87)
		Sedentary^d^	2.94 (1.97)	0.137	56	0.41 (−0.13, 0.94)	−0.42 (− 0.98, 0.13)
		LPA	**−1.88 (0.91)**	**0.041**	18	**−0.18 (− 0.35, − 0.01)**	**0.19 (0.01, 0.38)**
		MVPA	0.18 (0.27)	0.510	0.1	0.01 (−0.03, 0.06)	−0.02 (− 0.07, 0.03)
2 (*n* = 158)	15.1 (4.9)	Sleep	−0.07 (2.26)	0.977	69	−0.01 (− 0.74, 0.72)	0.01 (− 0.73, 0.75)
		Sedentary	0.39 (2.09)	0.852	46	0.05 (−0.46, 0.56)	−0.05 (− 0.59, 0.49)
		LPA	0.53 (2.06)	0.796	28	0.06 (−0.36, 0.48)	−0.06 (− 0.51, 0.39)
		MVPA	−0.86 (0.49)	0.084	0.6	−0.07 (− 0.15, 0.01)	0.08 (− 0.01, 0.17)
3.5 (*n* = 179)	15.5 (5.0)	Sleep	−0.05 (3.59)	0.988	65	−0.01 (−1.12, 1.10)	0.01 (−1.12, 1.14)
		Sedentary	−0.54 (2.94)	0.856	47	−0.07 (− 0.80, 0.66)	0.07 (− 0.69, 0.84)
		LPA	1.94 (2.10)	0.357	29	0.20 (−0.23, 0.64)	−0.22 (− 0.68, 0.25)
		MVPA	−1.35 (0.82)	0.102	2	−0.11 (− 0.25, 0.02)	0.13 (− 0.02, 0.27)
5 (*n* = 213)	15.7 (4.8)	Sleep	−0.29 (3.36)	0.932	64	−0.04 (−1.06, 0.97)	0.04 (− 0.99, 1.08)
		Sedentary	0.47 (2.50)	0.851	49	0.06 (−0.57, 0.69)	−0.06 (− 0.72, 0.60)
		LPA	0.41 (1.86)	0.825	28	0.04 (− 0.34, 0.42)	−0.05 (− 0.46, 0.36)
		MVPA	−0.60 (0.77)	0.438	3	−0.05 (− 0.18, 0.08)	0.06 (− 0.08, 0.20)

LPA is light physical activity, MVPA is moderate-to-vigorous physical activity

^a^Adjusted for randomised group, sex, primiparous, maternal education, household deprivation, ethnicity and maternal BMI. Analyses at 1 year of age further adjusted for whether the child was walking unaided or not walking

^b^Coefficient for the first isometric log ratio (ilr) co-ordinate, which is proportional to the log ratio of the behavior and the geometric mean of the remaining behaviors; adjusted for remaining ilr co-ordinates

^c^All sleep estimates include naps where relevant

^d^All sedentary estimates include wake after sleep onset

Bolded values indicate *P* < 0.05

In terms of bone health, higher levels of MVPA were consistently associated with higher TBLH BMD (Table 7) and TBLH BMC (Table 8). A 10% difference in MVPA at 2 to 5 years of age (0.6 to 3 min a day) produced statistically significant, albeit small, differences in TBLH BMD of 0.001 to 0.002 g/cm^2^ at the different ages. Very similar small effect sizes were observed in regards to MVPA and TBLH BMC (Table 8). Although a 10% increase in MVPA was associated with statistically significant differences in TBLH BMC, these values of 2.0 to 3.2 g would be considered negligible for practical purposes. No consistent relationships between other components of compositional time use and bone health were apparent (Tables 7 and 8).

**Table 7:** Predictive and concurrent relationships between sleep and activity components at 1, 2, 3.5 and 5 years of age and *total body minus head BMD* (g/cm^2^) at 5 years^a^

Age (years)	Mean BMD (SD) at 5 years of age (g/cm^2^)	Component	Regression coefficient^b^ (SE)	*p*-value	10% relative difference in minutes	Estimated difference (95% CI) in BMD with a 10% *increase* in component	Estimated difference (95% CI) in BMD with a 10% *decrease* in component
1 (*n* = 229)	0.608 (0.037)	Sleep^c^	0.010 (0.015)	0.516	71	0.002 (−0.003, 0.007)	−0.002 (− 0.007, 0.003)
		Sedentary^d^	**− 0.032 (0.015)**	**0.029**	56	**−0.004 (− 0.008, − 0.0005)**	**0.005 (0.0005, 0.009)**
		LPA	**0.024 (0.007)**	**0.001**	18	**0.002 (0.001, 0.004)**	**−0.002 (− 0.004, − 0.001)**
		MVPA	−0.001 (0.002)	0.632	0.1	0.000 (−0.0004, 0.0002)	0.000 (−0.0003, 0.0005)
2 (*n* = 158)	0.609 (0.036)	Sleep	−0.003 (0.015)	0.839	69	−0.001 (− 0.005, 0.004)	0.001 (− 0.004, 0.005)
		Sedentary	−0.010 (0.014)	0.465	46	−0.001 (− 0.005, 0.002)	0.001 (− 0.002, 0.005)
		LPA	0.002 (0.014)	0.904	28	0.000 (−0.003, 0.003)	0.000 (−0.003, 0.003)
		MVPA	**0.012 (0.003)**	**0.001**	0.6	**0.001 (0.0004, 0.0015)**	**−0.001 (− 0.0017, − 0.0005)**
3.5 (*n* = 178)	0.607 (0.037)	Sleep	−0.021 (0.027)	0.426	65	−0.003 (− 0.012, 0.005)	0.003 (− 0.005, 0.012)
		Sedentary	−0.007 (0.021)	0.751	47	−0.001 (− 0.006, 0.004)	0.001 (− 0.005, 0.006)
		LPA	0.015 (0.016)	0.351	29	0.002 (−0.002, 0.005)	−0.002 (− 0.005, 0.002)
		MVPA	**0.014 (0.006)**	**0.023**	2	**0.001 (0.0002, 0.0021)**	**−0.001 (− 0.0023, − 0.0002)**
5 (*n* = 213)	0.609 (0.037)	Sleep	**−0.054 (0.025)**	**0.031**	64	**−0.008 (− 0.0008, − 0.0159)**	**0.009 (0.0008, 0.0162)**
		Sedentary	0.036 (0.019)	0.055	49	0.005 (−0.0001, 0.0092)	−0.005 (− 0.0097, 0.0001)
		LPA	−0.001 (0.014)	0.953	28	0.000 (−0.003, 0.003)	0.000 (−0.003, 0.003)
		MVPA	**0.019 (0.006)**	**0.001**	3	**0.002 (0.0007, 0.0026)**	**−0.002 (− 0.0028, − 0.0007)**

LPA is light physical activity, MVPA is moderate-to-vigorous physical activity

^a^Adjusted for randomised group, sex, primiparous, maternal education, household deprivation, ethnicity and maternal BMI. Analyses at 1 year of age further adjusted for whether the child was walking unaided or not walking

^b^Coefficient for the first isometric log ratio (ilr) co-ordinate, which is proportional to the log ratio of the behavior and the geometric mean of the remaining behaviors; adjusted for remaining ilr co-ordinates

^c^All sleep estimates include naps where relevant

^d^All sedentary estimates include wake after sleep onset

Bolded values indicate *P* < 0.05

**Table 8:** Predictive relationships between sleep and activity components at 1, 2 and 3.5 years of age and *total body minus head BMC (g)* at 5 years^a^

Age (years)	Mean BMC (SD) at 5 years of age (g)	Component	Regression coefficient^b^ (SE)	*p*-value	10% relative difference in minutes	Estimated difference (95% CI) in BMC with a 10% *increase* in component	Estimated difference (95% CI) in BMC with a 10% *decrease* in component
1 (*n* = 229)	380 (74)	Sleep^c^	49.3 (27.5)	0.075	71	8.4 (−0.8, 17.6)	− 8.4 (− 17.7, 0.8)
		Sedentary^d^	**− 88.7 (27.7)**	**0.002**	56	**− 12.3 (− 19.8, − 4.8)**	**12.8 (5.0, 20.6)**
		LPA	**42.6 (12.8)**	**0.001**	18	**4.0 (1.7, 6.4)**	**−4.4 (− 7.0, − 1.8)**
		MVPA	−3.2 (3.8)	0.400	0.1	−0.3 (− 0.9, 0.4)	0.3 (− 0.4, 1.0)
2 (*n* = 158)	383 (73)	Sleep	19.6 (28.2)	0.488	69	3.2 (−5.9, 12.4)	−3.3 (−12.5, 6.0)
		Sedentary	−46.6 (26.2)	0.077	46	−5.8 (−12.2, 0.6)	6.1 (−0.6, 12.9)
		LPA	3.1 (25.7)	0.904	28	0.3 (−4.9, 5.6)	−0.4 (−6.0, 5.3)
		MVPA	**23.9 (6.1)**	**< 0.001**	0.6	**2.0 (1.0, 3.0)**	**−2.2 (−3.3, −1.1)**
3.5 (*n* = 178)	379 (74)	Sleep	−16.9 (50.4)	0.738	65	−2.7 (−18.3, 12.9)	2.7 (−13.2, 18.6)
		Sedentary	−26.0 (40.5)	0.522	47	−3.3 (−13.3, 6.7)	3.5 (−7.1, 14.0)
		LPA	16.9 (29.4)	0.565	29	1.8 (−4.3, 7.8)	−1.9 (−8.4, 4.6)
		MVPA	**26.0 (11.1)**	**0.021**	2	**2.2 (0.3, 4.0)**	**−2.4 (−4.4, −0.4)**
5 (*n* = 213)	383 (73)	Sleep	−93.5 (47.7)	0.051	64	−14.4 (−28.8, 0.005)	14.7 (−0.006, 29.4)
		Sedentary	**75.1 (35.4)**	**0.035**	49	**9.6 (0.7, 18.5)**	**−10.1 (−19.5, −0.8)**
		LPA	−19.6 (26.4)	0.458	28	−2.0 (−7.4, 3.3)	2.2 (−3.6, 8.0)
		MVPA	**38.0 (10.9)**	**0.001**	3	**3.2 (1.4, 5.0)**	**−3.5 (−5.5, −1.6)**

LPA is light physical activity, MVPA is moderate-to-vigorous physical activity

^a^Adjusted for randomised group, sex, primiparous, maternal education, household deprivation, ethnicity and maternal BMI. Analyses at 1 year of age further adjusted for whether the child was walking unaided or not walking

^b^Coefficient for the first isometric log ratio (ilr) co-ordinate, which is proportional to the log ratio of the behavior and the geometric mean of the remaining behaviors; adjusted for remaining ilr co-ordinates

^c^All sleep estimates include naps where relevant

^d^All sedentary estimates include wake after sleep onset

Bolded values indicate *P* < 0.05

## Discussion

Our data demonstrate that considerable changes in compositional time use occur across the 24-h day over the preschool years. In late infancy, children are spending just under 12 h asleep on average, about 3 h in activity of light intensity, and the majority of awake time in sedentary pastimes. By 5 years of age, substantial increases in light-intensity activity occur, as do increases in MVPA, with corresponding reductions in time spent asleep, particularly as naps during the day. However, compositional time use showed little relationship to body composition, whether assessed cross-sectionally, prospectively, or longitudinally. Spending more time asleep or less time in LPA or sedentary activity were associated cross-sectionally with lower BMI z-scores at 3.5 years of age, but significant relationships were not observed at any other age or longitudinally. Although higher time spent in MVPA was positively associated with bone health, the actual differences observed were very small and unlikely to be of clinical significance. In combination, although observational, these findings would provide little support for compositional time use being associated with body composition in young children.

The degree to which sedentary behavior, physical activity, and/or sleep influence body composition and bone health in young preschool-aged children is uncertain. A series of recent comprehensive systematic reviews have highlighted that while objectively measured sedentary behavior appears to have little association with adiposity or bone health [37], the majority of studies support an adverse effect of shortened sleep on weight status [38]. In terms of physical activity, findings are more mixed. Although time spent in MVPA has frequently been associated with reduced adiposity and improved bone health in observational research, meta-analyses of the few intervention studies did not show any difference in BMI according to activity levels [39]. Moreover, time in light and moderate intensity activity was not consistently associated with favourable health outcomes [39]. As highlighted in these reviews, the risk of bias is high for much of this evidence, leading to overall gradings of low or very low for the quality of the evidence, according to GRADE frameworks [40]. These mixed findings help illustrate the importance of compositional analyses, particularly when others have shown that omitting individual behaviors (such as sleep) from models can substantially affect the interpretation of the relationships between the remaining behaviors and BMI, including changing the direction of the relationships [12].

It is difficult to compare our findings with the literature given the scarcity of compositional time-use studies, particularly in preschool children. Studies in older children and adolescents are all cross-sectional and have generally provided estimates for the effect on BMI z-score of reallocating time in one activity to another activity. For example, in one study in 10-year old children, the predicted difference in BMI z-score if 15 min of sleep is replaced with 15 min of MVPA per day is − 0.48. Interestingly, the effect is not linear, that is, doing 15 min per day less MVPA to gain 15 min of sleep results in a predicted difference in BMI z-score of 0.88, almost twice that of the reverse direction [11]. Others have also demonstrated seemingly substantial benefits to BMI of substituting sedentary behavior for MVPA; replacing one hour of sedentary time with the same amount of MVPA was associated with a difference in BMI of up to 1.4 kg/m^2^ across a wide age range (5–19 years) [13]. These differences in BMI are substantial, and upon initial inspection provide significant support for an important impact of MVPA on BMI in children. However, these studies hypothesize a somewhat unrealistic substitution scenario where time spent in one behavior would be entirely reallocated to one other behavior with no effect on the remaining behaviors, ignoring the co-dependent nature of the behaviors. The biggest differences are consistently seen when substituting a number of minutes for MVPA. This is because MVPA, while possibly having an influence on body composition, is the smallest component in the day, where a 15 min reallocation is, for example, 57% of mean MVPA time but only 3% of mean sleep time [11]. Therefore it isn’t the small, insignificant reallocation of sleep that contributes to the estimated difference in BMI z-score but rather the substantial reallocation to MVPA. However, presenting estimates in this way can give a misleading view of what behaviors should be encouraged and discouraged. We have attempted to present associations between time-use and body composition in a more transparent and realistic way, as described by Dumuid and colleagues [41], by reporting on the reallocation of a *proportion* of the component (not a set number of minutes) from one activity to *all others* [41].

The single existing compositional time-use study in preschool aged children reported that overall time use was associated with BMI z-score in 3–4 year old children, but that significant effects were not apparent for any of the individual components of time use in cross-sectional analyses [10]. Although the present study demonstrated that sleep was inversely associated, and sedentary and LPA positively associated with BMI, this was not a consistent finding. Our data clearly show that significant relationships between time use and BMI were only observed at one of the four cross-sectional time points (3.5 years of age). Importantly, compositional time use did not predict BMI at any of the longitudinal time points examined. Discrepancy in findings may be due to the use of questionnaires [10] versus actigraphy (current study) to assess sleep, and/or a result of different cutpoints to denote intensity of activity between studies. Use of different processing criteria is a well-known limitation of accelerometry analyses which limits direct comparison between studies [42]. Regardless of these issues, our analyses clearly demonstrate that although time use might be related to BMI cross-sectionally, no such relationships are apparent in longitudinal analyses, at least in this young cohort.

Our findings also indicate that variation in time use has no relationship with percentage body fat at this young age. We cannot compare our findings with the literature as no studies appear to have examined compositional time use over the full 24-h period to evaluate relationships with percent fat in children [3]. Replacing sedentary time with MVPA has been associated with lower adiposity in the few existing studies in children that have used isotemporal analyses to determine how reallocation of time use influences adiposity [43, 44]. However, none of the studies included had measures of sleep and thus 24-h time use. Work from our group demonstrates that correctly accounting for sleep is crucial for obtaining accurate measures of sedentary time; estimates of sedentary time across a 24-h day ranged from 556 to 1145 min depending on the sleep scoring method utilised [26].

Engaging in sufficient moderate-to-vigorous physical activity is important for bone development [37], even in young children [45], and limited work suggests that sufficient sleep might also be important for bone [46]. However, no studies have examined combinations of physical activity, sedentary behavior, and sleep in relation to BMC or BMD in children [3]. Although our study consistently demonstrated a protective effect of MVPA at 2, 3.5 and 5 years of age on bone outcomes at 5 years of age, these differences were small, and unlikely to be clinically relevant as they fell within the expected error for repeat scans on children (0.004 g/cm^2^ or 0.6% CV). By contrast, our findings regarding the impact of sleep and sedentary findings were much less consistent and thus should be interpreted with caution. Time spent asleep was not related to bone health, with a single exception (sleep negatively impacting on BMD at 5 years of age in cross-sectional analyses), whereas sedentary time at one year of age was positively, and time at 5 years of age negatively, associated with bone outcomes at 5 years. Our findings agree with recent reviews that have highlighted little effect of sedentary time or sleep on bone in young children [46, 47]. It is feasible that stronger relationships might be observed in older children given differences in bone accrual with maturation; children undergoing puberty gain bone more rapidly [48], perhaps allowing greater influence of relevant behaviors such as sleep and physical activity.

Our study has several strengths including repeated assessment of measures of interest, the use of 24-h accelerometry rather than a combination of approaches to measure all behaviors of interest, and the inclusion of DXA, allowing investigation of relationships with body composition and bone health, as well as BMI. However, our study also has some limitations. Because we were interested in sleep and how it might impact on other components of the day as well as more broad relationships with health, each 24-h ‘day’ is calculated as the time the child woke up on day 1 until the time they woke up on day 2 (and so on). This means that the length of each day varies both within and between participants, requiring all data to be scaled to 24 h periods. However, this method should more appropriately capture the co-dependence of the sleep and activity components with repeated days of measurement and it is the structure of the day, not the actual time, that is relevant [7]. Second, difficulty remains in determining the most appropriate cutpoints to denote different intensities of activity, particularly across different age groups [42]. Commonly accepted cutpoints for the Actical accelerometer do not exist for children spanning the age range in this study (1 to 5 years), which is problematic given the rapid development that occurs over this time [42]. We chose cutpoints from a variety of sources [33, 34] and applied them across the age range to allow comparisons by age, but this may have introduced some error, particularly in those under three years of age and for determining MVPA. These cutpoints were derived using an epoch length of 15 s, which may be too long to correctly capture all of the brief bouts of MVPA that inherently occur via the intermittent nature of play in very young children [49, 50]. As others have shown, shorter epochs (5 s) provide improved estimates of sedentary behavior and physical activity in 2–3 year old children using the ActiGraph accelerometer [51]. The cut-point used to denote MVPA in the current study (≥ 698 counts per 15 s) was based on validation studies in toddlers and pre-schoolers [32, 33] but is also considerably higher than other validation studies published subsequently [34, 52]. Our findings regarding MVPA should thus be viewed with caution, particularly given others have stressed the difficulties of assessing MVPA in those under three years of age [51]. In our analyses, we controlled for sex rather than undertaking sex-specific analyses so that our sample sizes, and therefore power, were not reduced. This may be a limitation given that differences in physical activity may be apparent even at this young age. Sensitivity analyses did demonstrate that the significant cross-sectional relationships observed between compositional time use and BMI z-score at 3.5 years of age were only apparent in girls (data not shown). However, because of the lack of significant relationships observed generally in our study, and the small effect sizes in those that were significant, we did not complete any remaining sex-specific analyses. Although several methods have been suggested for adjusting BMC or BMD for factors such as body size, pubertal stage, skeletal maturity and body composition, there is currently no consensus on the optimum method to use [53]. The International Society for Clinical Densitometry suggests adjusting for height is appropriate in children with chronic diseases in whom poor growth and delayed puberty adversely affect bone size [54]. As our participants were healthy, and fewer than 5% of the cohort had height Z-scores <3rd or > 97th percentile, we considered that adjusting for BMI Z-score was more appropriate. We only had valid accelerometry data suitable for these analyses from 27 to 45% of the original cohort at any one time, and only 139 children (17.3% of the original cohort) had accelerometry data at all 4 time points, introducing the possibility that our results might not generalise to the population as a whole. However, comparison of demographic variables at baseline in those who did and did not supply data showed relatively little difference between the groups. Finally, it was not possible to determine the effect of compositional time use on body composition at 5 years of age with adjustment for body composition at a younger age as we only had DXA measurements at 5 years of age. Adjustment for other variables related to body composition such as dietary intake and screen use was also not possible as consistent measures of these variables were not available throughout the project.

## Conclusions

Considerable changes in time use occur across the 24-h day from infancy to 5 years of age. However, how these young children used their time showed few significant relationships with body composition and bone health at this young age using analyses appropriately accounting for compositional time use.

## Additional file

Additional file 1: **Table S1.** Variation matrices^1^ between sleep and activity components at 1, 2, 3.5 and 5 years of age. (DOCX 28 kb)

## Abbreviations

BMC: Bone mineral content
BMD: Bone mineral density
BMI: Body mass index
DXA: Dual-energy x-ray absorptiometry
LPA: Light physical activity
MVPA: Moderate-to-vigorous physical activity
TBLH: Total body less head
WASO: Wake after sleep onset
