# Supplementary material for: 24-h movement behaviors from infancy to preschool: cross-sectional and longitudinal relationships with body composition and bone health
Source: Int J Behav Nutr Phys Act. 2018 Nov 26;15:118. doi: 10.1186/s12966-018-0753-6 (PMC6260686; doi:10.1186/s12966-018-0753-6)
Supplement: Supplementary file 1 — Table S1. Variation matrices1 between sleep and activity components at 1, 2, 3.5 and 5 years of age. (DOCX 28 kb) [file 12966_2018_753_MOESM1_ESM.docx]

**Additional file 1**

**Table S1**.Variation matrices^1^ between sleep and activity components at 1, 2, 3.5 and 5 years of age.

|  | Age (years) | Sleep | Sedentary | LPA |
| --- | --- | --- | --- | --- |
| Sedentary | 1 | 0.03 | 0 |  |
|  | 2 | 0.04 | 0 |  |
|  | 3.5 | 0.02 | 0 |  |
|  | 5 | 0.02 | 0 |  |
| LPA | 1 | 0.19 | 0.20 | 0 |
|  | 2 | 0.06 | 0.08 | 0 |
|  | 3.5 | 0.05 | 0.08 | 0 |
|  | 5 | 0.05 | 0.08 | 0 |
| MVPA | 1 | 1.58 | 1.58 | 1.41 |
|  | 2 | 1.00 | 1.06 | 0.87 |
|  | 3.5 | 0.35 | 0.41 | 0.26 |
|  | 5 | 0.26 | 0.32 | 0.21 |

^1^A variation matrix is the matrix of the variances of the pair-wise log ratios for the components. This is a measure of proportionality or co-dependence in that values close to zero have high proportionality/co-dependence and higher values have lower proportionality/co-dependence.
